# Synergistic Adsorption–Enhancement of Bamboo–Aramid Fibers in SMA-13 Asphalt Mixtures

**DOI:** 10.3390/ma19132746

**Published:** 2026-06-26

**Authors:** Yingying Zhou, Yanping Sheng, Huilin Wang, Xiaoting Wang, Zhaofeng Xue, Bohan Sheng

**Affiliations:** 1 School of Materials Science and Engineering, Chang’an University, Xi’an 710061, China; 18996475079@163.com (Y.Z.); wangxiaoting1023@163.com (X.W.); shb860328@163.com (B.S.); 2 Qinghai Transportation Science Research Institute, Xining 810003, China

**Keywords:** SMA-13 asphalt mixture, bamboo fiber, aramid fiber, hybrid fiber modification, pavement performance

## Abstract

The synergistic use of natural bamboo fiber and synthetic aramid fiber in asphalt mixtures has received limited research attention, particularly regarding the optimal blending ratio under a constant total fiber content and the underlying reinforcement mechanisms. This study systematically investigated the co-blending of bamboo and aramid fibers in SMA-13 asphalt mixtures with a fixed total fiber content of 0.3%. Five mixture groups were prepared, LF (0.3% lignin fiber, control), BF (0.3% bamboo fiber), as well as three hybrid groups: ABF-1 (0.27% bamboo + 0.03% aramid, 9:1), ABF-2 (0.24% bamboo + 0.06% aramid, 4:1), and ABF-3 (0.21% bamboo + 0.09% aramid, 7:3). The mixtures were evaluated using rutting tests, low-temperature flexural beam tests, moisture stability tests, and AMPT dynamic modulus testing. The results demonstrate that hybrid-fiber mixtures outperform single-fiber mixtures, with ABF-2 exhibiting the best overall performance. Compared with LF and BF, ABF-2 achieved a dynamic stability of 6921 passes/mm (increases of 97.7% and 52.7%, respectively); flexural tensile strength increased by 43.1% and 32.1%; maximum flexural tensile strain increased by 42.6% and 35.0%; and retained stability improved by 10.8% and 12.5%. AMPT results indicated a higher dynamic modulus and lower phase angle for the hybrid system, suggesting an enhanced elastic response. The superior performance of ABF-2 is attributed to the complementary adsorption–stabilization effect of bamboo fiber and bridging–reinforcement effect of aramid fiber. This study provides quantitative evidence for the beneficial combination of natural and synthetic fibers in asphalt mixtures and identifies key limitations that warrant future investigation.

## 1. Introduction

Stone Mastic Asphalt (SMA) is a gap-graded asphalt mixture in which fibers are commonly used as stabilizers to improve mixture stability [1,2,3,4]. To further enhance the overall pavement performance of SMA mixtures, researchers have explored the addition of various fiber materials. Among them, bamboo fiber and aramid fiber, both with distinctive properties, have attracted increasing attention [5,6,7,8]. As a natural material, bamboo fiber exhibits good adsorption capacity and reinforcing effects, thereby enhancing the adhesion of the asphalt binder, resistance to moisture damage, and resistance to cracking in asphalt mixtures [9,10]. Aramid fiber provides high strength and high-temperature resistance, offering benefits in rutting and fatigue performance and cracking resistance, although its high cost limits practical application [11,12,13,14]. Recently, increasing studies have focused on the synergistic use of bamboo fiber and aramid fiber, showing that their combined application can further enhance pavement performance [15,16,17].

In recent years, bamboo fiber has attracted increasing attention as an environmentally compatible additive for asphalt mixtures. Sheng et al. [18] investigated the role of bamboo fiber in asphalt mixtures with different gradations and reported that it improves overall mixture performance, showing superior pavement performance compared with other plant fibers. Jia et al. [19] indicated that the mechanical properties are optimized at a bamboo fiber content of 0.3 percent, accompanied by an increase in dynamic modulus, thereby improving high-temperature shear resistance and low-temperature flexibility. Cao et al. [20] found that bamboo fiber enhances high-temperature stability, low-temperature cracking resistance, moisture resistance, and aging resistance, and it outperforms lignin fiber in overall performance. The improvement in aging resistance is commonly attributed to the strong asphalt adsorption and stabilizing action of bamboo fibers, which reduces the amount of free asphalt and helps maintain a more stable mastic structure during oxidative aging, thereby mitigating stiffness growth and fatigue deterioration. Jia et al. [21] further confirmed the anti-aging capability of bamboo fiber by suppressing stiffness growth and mitigating fatigue deterioration. Li et al. [22,23] improved the adhesion between bamboo fiber and asphalt by applying alkaline treatment combined with coupling agents, thereby enhancing performance at both high and low temperatures and clarifying the interfacial strengthening mechanism. From a review perspective, Zheng et al. [24] summarized the reinforcement mechanisms, modification methods, and application challenges of bamboo fiber, and outlined future research directions.

Aramid fiber has been increasingly investigated as a modifier for improving asphalt mixture performance due to its high strength and high-temperature resistance. Saeed S. Saliani et al. [25] reported that incorporating aramid fiber at a dosage of 0.05 percent improves fatigue performance while maintaining relatively stable viscoelastic behavior after long-term aging. Saeed Badeli et al. [26] verified durability advantages through freeze–thaw cycling and dynamic modulus testing, indicating improved resistance to moisture damage and performance deterioration induced by temperature variations. Ali Raza Khan et al. [27] found, based on dynamic modulus and fatigue tests, that aramid fiber reduces aging sensitivity and delays performance degradation, and Jia et al. [28] further confirmed that aramid fiber mitigates fatigue deterioration under different aging durations and identified four days of aging as a critical stage of fatigue decline. He et al. [29] proposed quantitative indices to characterize agglomeration and showed that agglomeration is strongly affected by mixing time, temperature, and other conditions, highlighting the need to suppress agglomeration through process control to ensure mixture performance. Wisniewski et al. [30] further showed that aramid fiber can effectively reduce fatigue damage in asphalt mixtures, providing support for its application under heavy traffic loads and aged pavement conditions.

As summarized in Table 1, bamboo fiber and aramid fiber offer distinct and complementary advantages. Bamboo fiber excels in adsorbing free asphalt and stabilizing the asphalt mastic, which enhances high-temperature stability and moisture resistance. However, its relatively low tensile strength (600 MPa, Table 2) limits its ability to resist crack propagation under tensile loading. In contrast, aramid fiber provides high tensile strength (2900 MPa, Table 2) and excellent thermal stability, making it effective in delaying fatigue crack initiation and improving dynamic modulus. Nevertheless, its high cost and tendency to agglomerate at high concentrations constrain its practical application. The combination of these two fibers in a hybrid system offers the potential to achieve a balanced improvement in multiple performance dimensions while controlling material cost.

Previous studies have shown that the effectiveness of fiber reinforcement depends not only on fiber type and dosage, but also on fiber dispersion, interfacial bonding, and the resulting mastic stability and load transfer capability within the aggregate skeleton [31,32]. Therefore, hybrid fiber blending is increasingly adopted to combine complementary mechanisms and to achieve a more balanced improvement in high-temperature stability, cracking resistance, and moisture durability [33].

Compared with single-fiber modification, blending different types of fibers is more effective in improving the overall pavement performance of asphalt mixtures, particularly in terms of high-temperature stability, low-temperature cracking resistance, and resistance to moisture-induced damage. Guo et al. [34] systematically reviewed the various applications of different fiber types in asphalt pavements and discussed the main variables governing fiber effectiveness as well as the associated reinforcing mechanisms. Ali Raza Khan et al. [27] investigated the synergistic effect of co-blending aramid fiber and synthetic aramid fiber, and reported that this combination increases the dynamic modulus and delays fatigue-crack initiation. Xu et al. [35] evaluated freeze–thaw damage through laboratory testing combined with discrete element modeling, confirming that aramid–lignin fiber blends can enhance the structural durability of the mixture and improve freeze–thaw resistance. In addition, Yang et al. [36] emphasized that uniform fiber distribution is a key factor for improving structural stability and extending the crack-resistant service life. Overall, hybrid fiber blending can exploit complementary advantages and generate synergistic reinforcement, showing strong potential for improving the comprehensive durability and engineering applicability of asphalt mixtures.

Despite growing research interest in the use of hybrid fiber modification for asphalt mixtures, several critical knowledge gaps remain unaddressed. First, most existing studies have focused on the blending of synthetic fibers or the combination of synthetic fibers with lignin fibers; however, the synergistic interplay between natural bamboo fiber, which possesses strong asphalt adsorption capacity, and aramid fiber, characterized by high strength and modulus, has rarely been investigated quantitatively. Second, within the bamboo–aramid hybrid system, the optimal mixing ratio under a fixed total fiber content has yet to be systematically determined, leading to uncertainty regarding the balance between the adsorption effect and the reinforcement effect.

To address these gaps, this study systematically evaluated the high-temperature stability, low-temperature crack resistance, moisture stability, and viscoelastic properties of bamboo–aramid hybrid fiber-reinforced SMA-13 mixtures under a constant total fiber content of 0.3%, and determined the optimal bamboo-to-aramid mixing ratio among three candidate proportions (9:1, 4:1, and 7:3). The results indicate that the hybrid fiber exhibits superior overall performance compared to single fibers and other control groups, thereby providing a reference for future research and engineering applications.

## 2. Materials and Methods

### 2.1. Materials

#### 2.1.1. Fiber

**Figure 1:**
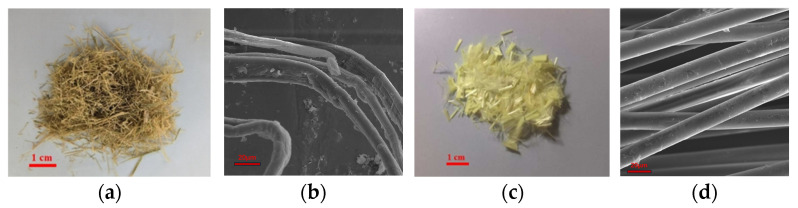
Appearance and Microscopic Morphology of Bamboo and Aramid Fibers: (**a**) appearance of bamboo fiber; (**b**) microscopic morphology of bamboo fiber; (**c**) appearance of aramid fiber; (**d**) microscopic morphology of aramid fiber.

In this study, bamboo fiber and aramid fiber were used, and their appearances are shown in Figure 1, while the key physical parameters are summarized in Table 2. The bamboo fibers were obtained from moso bamboo native to Zhejiang Province (Hangzhou, China); due to the lack of screening, the fibers exhibit non-uniform lengths and a rough surface, with a slight tendency to agglomerate. The aramid fiber is a para-aramid fiber manufactured by DuPont (Wilmington, DE, USA), featuring high strength and modulus as well as good thermal and chemical stability.

SEM observations indicate that the bamboo fibers have an irregular surface with abundant pores, which provide space for asphalt absorption and thus result in a relatively high oil absorption rate. In contrast, the aramid fiber has a relatively straight and smooth surface, which helps it maintain a stable structure within the asphalt–aggregate skeleton and enhances the mechanical integrity of the system. All fiber-related indices were tested in accordance with the technical standard “Fibers for Asphalt Pavement” (JT/T 533-2020) [37].

#### 2.1.2. Aggregate

In this study, basalt aggregates from Xi’an were employed. The slag powder producer is the second branch of Dezhou Guangjian Building Materials Co., Ltd. (Dezhou, China). The properties of both coarse and fine aggregates were determined in compliance with the Test Methods of Aggregates for Highway Engineering (JTG 3432-2024) [38], and the main test results are listed in Table 3, Table 4 and Table 5. All results were within the specified limits, indicating that the selected aggregates met the required performance standards for use in asphalt mixtures.

#### 2.1.3. Asphalt

The fundamental properties of the SBS-modified asphalt (Deda Transportation Construction Development Group Co., Ltd., Dezhou City, China) were measured in compliance with the Standard Test Methods of Asphalt and Asphalt Mixture for Highway Engineering (JTG 3410-2025) [39], and the corresponding results are listed in Table 6.

### 2.2. Mix Proportion Design

#### 2.2.1. Gradation Composition

**Figure 2:**
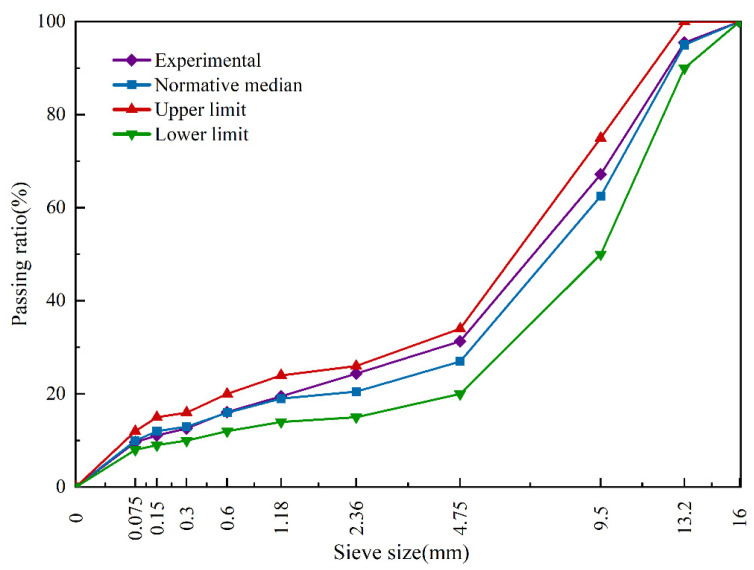
Gradation Curve of SMA-13.

The gradation curve used in this study (Figure 2) is generally centered around the median gradation specified for SMA-13, but with a slightly increased proportion of fine aggregates compared to the median value. This adjustment was made for the following technical reasons: the fibers used in this study are shorter than 6 mm, and a higher content of fine aggregates enhances the fiber–aggregate interlocking effect. The increased fine aggregate fraction provides more contact points for fiber anchorage, facilitating the development of a stable three-dimensional fiber network within the mixture. This gradation modification was determined based on pre-trial tests. It should be noted that all volumetric parameters of the selected gradation still fall within the specification limits, ensuring compliance with standard requirements.

**Figure 3:**
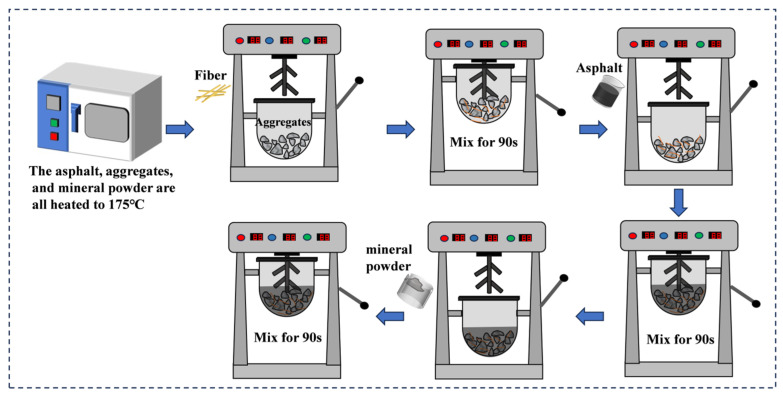
Schematic of the Mixing Process of Fiber-Reinforced Asphalt Mixtures.

Dry-mixing and wet-mixing are two common approaches for introducing fibers into asphalt mixtures. In dry-mixing, fibers are blended with aggregates first and then mixed with asphalt, whereas in wet-mixing, the fibers are pre-mixed with asphalt to form a fiber–asphalt mastic before being combined with aggregates [40,41,42]. Considering field practicality and the requirement for adequate fiber dispersion, the dry-mixing method was adopted in this study for bamboo– and aramid–fiber-reinforced SMA mixtures. The mixing procedure is schematically illustrated in Figure 3.

This work investigates the optimal blending ratio between bamboo fiber and aramid fiber in fiber-modified asphalt mixtures by setting different hybrid proportions. The objective is to assess the pavement-related performance and mechanical properties of the SMA mixture at the optimal fiber-blending ratio. To this end, three experimental groups with different hybrid ratios were designed, and two control groups were established for comparative analysis. The specific fiber blending ratios and the naming of each test group are shown in Table 7.

The total fiber content was fixed at 0.3% by the total mass of the mixture. This value was selected based on pre-trial tests conducted in our laboratory, which indicated that 0.3% bamboo fiber alone provided optimal performance in SMA mixtures, consistent with findings by Jia et al. [19], and the recommended aramid fiber dosage range of 0.05–0.1% reported by Saliani et al. [25] and Badeli et al. [26]. Fixing the total fiber content at 0.3% allows for a systematic comparison of different bamboo-to-aramid blending ratios without introducing variability from different total fiber dosages. Three blending ratios were designed: 9:1, 4:1, and 7:3. These ratios were selected to cover the transition from bamboo-dominated adsorption effects to aramid-dominated reinforcement effects while maintaining a constant total fiber content. Pre-trial tests indicated that aramid fiber contents exceeding 0.1% (i.e., >1/3 of total fiber mass) led to noticeable agglomeration and handling difficulties; therefore, the maximum aramid content was set at 0.09%.

A key feature of this study is the experimental design that allows for quantitative separation of the contributions of bamboo and aramid fibers. Specifically, comparing BF with LF isolates the effect of replacing lignin fiber with bamboo fiber. Comparing ABF with BF isolates the additional contribution of adding 0.06% aramid fiber while reducing bamboo fiber content by 0.06%. Thus, any performance difference between ABF-2 and BF can be attributed to the introduction of aramid fiber and the associated hybrid interaction. The purpose of comparing ABF and BF is to examine the differences in the performance of asphalt mixtures under different fiber blending ratios and the interactions among blended fibers.

#### 2.2.2. Determination of Optimum Asphalt–Aggregate Ratio

The SMA mixtures with different hybrid fiber ratios were prepared using the mixing method described above. The hybrid fiber SMA-13 mixtures were compacted using a Marshall compactor (Model LR-T0702A, produced by Nanjing Lianrui Technology Co., Ltd., Nanjing, China) under double-sided compaction, with 75 blows applied to each side. Five specimens were fabricated for each group as described above. The optimum asphalt–aggregate ratio was established in compliance with the Technical Specification for Construction of Highway Asphalt Pavement (JTG F40-2023) [43].

Taking as an example the mixture containing 0.09% aramid fiber and 0.021% bamboo fiber, four levels of the asphalt–aggregate ratio—5.5%, 6.0%, 6.5%, and 7.0%—were selected, as specified in the Technical Specifications for Highway Asphalt Pavement Construction (JTG F40-2023), to fabricate Marshall specimens of hybrid fiber-modified SMA mixtures. The volumetric parameters, Marshall stability, flow values, and other relevant indices were then measured and calculated, and the corresponding data are listed in Table 8.

The asphalt–aggregate ratios associated with the maximum bulk density, the maximum Marshall stability, the median air void content, and the median asphalt saturation are denoted a_1_, a_2_, a_3_, and a_4_, respectively.

Taking the ABF-3 mixture as an example, four asphalt–aggregate ratios—5.5%, 6.0%, 6.5%, and 7.0%—were selected according to JTG F40-2023. Marshall specimens were prepared for each ratio, and the volumetric properties and Marshall stability were measured. The optimum asphalt content (OAC) was determined using a two-step approach:

Step 1—Calculation of OAC_1_: Four candidate asphalt contents were identified as follows:

a_1_ = asphalt content corresponding to maximum bulk density: (6.5%);

a_2_ = asphalt content corresponding to maximum Marshall stability: (6.5%);

a_3_ = asphalt content corresponding to the median air void content: (6.5 + 6.0)/2 = 6.25%;

a_4_ = asphalt content corresponding to the median asphalt saturation: (6.5 + 6.0)/2 = 6.25%.

Then, OAC_1_ = (a_1_ + a_2_ + a_3_ + a_4_)/4 = (6.5 + 6.5 + 6.25 + 6.25)/4 = 6.375%.

Step 2—Calculation of OAC_2_: The range of asphalt contents that satisfy all specification requirements was determined. From Table 7, the lower bound (${OAC}_{min}$) is 5.5% and the upper bound (${OAC}_{max}$) is 7.5%. Thus, ${OAC}_{2}$ = (${OAC}_{min}$ + ${OAC}_{max}$)/2 = (5.5 + 7.5)/2 = 6.5%.
Step 3—Final optimum asphalt content: OAC = (${OAC}_{1}$ + ${OAC}_{2}$)/2 = (6.5 + 6.375)/2 = 6.3125% ≈ 6.3%.

The optimal asphalt contents for the remaining groups were obtained by the same procedure, and the results are listed in Table 9, from which it can be seen that the optimal asphalt contents for the five SMA mixtures are 6.6%, 6.4%, 6.6%, 6.5%, and 6.3%, respectively.

#### 2.2.3. Asphalt Content Test

Once the optimum asphalt content of the fiber-reinforced SMA mixture has been determined, it is necessary to verify that the resulting mixture satisfies the specified requirements. In this study, the Schellenberg binder drainage test and the Cantabro loss test were carried out to evaluate the suitability of the selected asphalt content. The detailed results are presented in Table 10 and Table 11.

The drainage loss of asphalt is determined using Equation (1):

(1) $$∆m={\;(m}_{2}-m_{0})/\;(m_{1}-m_{0})$$

$m_{0}$—mass of the beaker alone (g);

$m_{1}$—combined mass of the beaker and asphalt mixture (g);

$m_{2}$—mass of the beaker together with the drained asphalt mixture, fine aggregates, and mastic (g);

∆m—percentage of asphalt drainage loss (%).

Based on the test results, the asphalt contents of the SMA mixtures containing lignin fibers, bamboo fibers, and hybrid fibers all satisfy the specified requirements.

### 2.3. Test Methods

All tests were conducted with at least three replicate specimens for each mixture group. For each test, the mean value and standard deviation (SD) were calculated. Error bars in all figures represent ±1 SD. For Marshall stability and flow values, individual specimen measurements are reported, and the mean ± SD is presented in the tables.

#### 2.3.1. High-Temperature Performance Test

**Figure 4:**
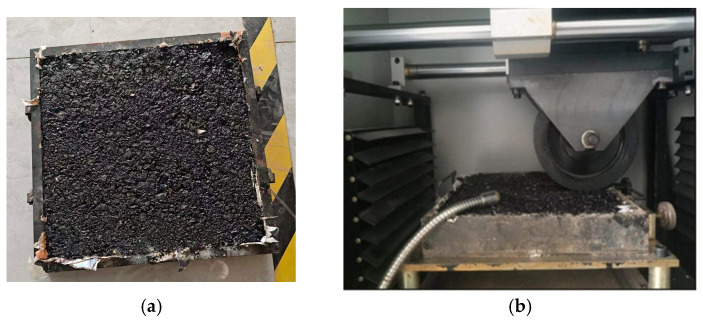
Rutting Test of Fiber-Reinforced Asphalt Mixtures: (**a**) Asphalt Mixture Rutting Slab. (**b**) Rutting Test Procedure.

In compliance with the Standard Test Methods of Asphalt and Asphalt Mixture for Highway Engineering (JTG 3410-2025), hybrid fiber SMA slabs (300 mm × 300 mm × 50 mm) were prepared using a wheel compactor (HYCX-1* model, produced by Hebei Dahong Experimental Instruments Co., Ltd., Cangzhou, China), as shown in Figure 4a. Before the rutting test, the slabs and the rutting mold were preconditioned in an oven at 60 °C for a minimum of 5 h to ensure a uniform temperature distribution throughout. After conditioning, the specimens were inserted into the wheel-tracking testing machine (JTC2-1 model, produced by Beijing Jietong Technology Co., Ltd., Beijing, China), with wheel position and sensors carefully adjusted to align the wheel rolling direction with the rutting formation direction. During the test, the temperature was monitored in real time, and an automatic deformation recorder was employed to simulate one hour of rolling traffic load. Dynamic stability of the hybrid fiber SMA mixture was evaluated based on the rut depths recorded at 45 and 60 min, as shown in Figure 4b.

Dynamic stability of the hybrid fiber SMA mixture is determined using Equation (2):

(2) $$DS=\left(t_{2}-t_{1}\right)\times \frac{N}{d_{2}-d_{1}}\times C_{1}\times C_{2}$$

*DS*—dynamic stability of the asphalt mixture (cycles/mm);

*d*_1_—deformation at time *t*_1_;

*d*_2_—deformation at time *t*_2_;

*C*_1_—correction factor for the type of testing machine;

*C*_2_—correction factor for the specimen;

*N*—reciprocating speed of the test wheel, typically 42 cycles/min.

#### 2.3.2. Low-Temperature Cracking Resistance Test

**Figure 5:**
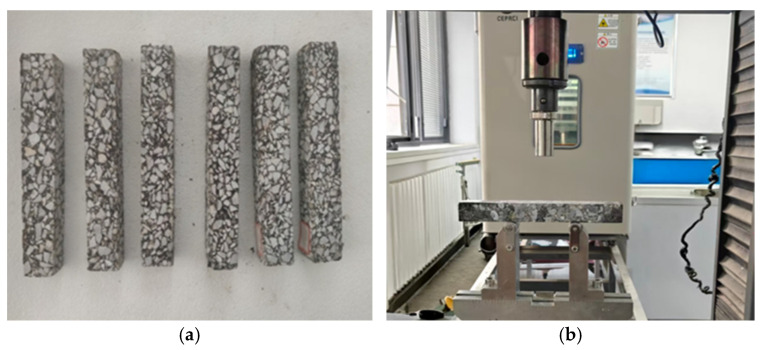
Beam Specimens of Fiber-Reinforced Asphalt Mixtures: (**a**) Beam Specimens of Asphalt Mixtures. (**b**) Low-Temperature Crack Resistance Test.

According to the requirements of Standard Test Methods of Asphalt and Asphalt Mixture for Highway Engineering (JTG 3410-2025) T 0703-2025 in the Test Regulations, beam specimens of the hybrid fiber-reinforced SMA mixture were cut from the rutting slabs using a dedicated sawing machine. The resulting beams had nominal dimensions of 250 mm × 30 mm × 35 mm and are shown in Figure 5a. Prior to testing, the beam specimens were conditioned in a temperature-controlled chamber at −10 °C for 6 h. After conditioning, the fixture of the universal testing machine (Model CMT5305, made by MTS Systems (China) Co., Ltd., Shanghai, China) was adjusted to provide a span of 200 mm between the two supports. A loading rate of 50 mm/min was applied during the test, as depicted in Figure 5b. During loading, the load–displacement response was continuously recorded. Based on the recorded data, key indices such as flexural tensile strength and maximum bending strain were derived to evaluate the low-temperature cracking performance of the hybrid fiber-reinforced SMA mixture.

Low-temperature flexural beam testing was employed to evaluate the cracking performance of the SMA mixture. Based on Equations (3)–(5), the following failure parameters were calculated: the flexural tensile strength $R_{B}$ (MPa), the maximum tensile strain at the bottom of the beam $\varepsilon_{B}\;(\mu \varepsilon$) and the flexural stiffness modulus $S_{B}$ (MPa):

(3) $$R_{B}=\frac{3LP_{B}}{2bh^{2}}$$

(4) $$\varepsilon_{B}=\frac{6hd}{L^{2}}$$

(5) $$S_{B}=\frac{R_{B}}{\varepsilon_{B}}$$

*b*—beam width at mid-span (mm);

*h*—beam height at mid-span (mm);

*L*—span length of the beam (mm);

*P_B_*—maximum failure load (N);

*d*—mid-span deflection at failure (mm).

#### 2.3.3. Moisture Stability Test

To simulate the moisture damage mechanism of asphalt mixtures, two laboratory tests were employed to evaluate the moisture stability of fiber-reinforced SMA mixtures, namely the Immersed Marshall test and the Freeze–Thaw Splitting test. In compliance with the Standard Test Methods of Asphalt and Asphalt Mixture for Highway Engineering (JTG 3410-2025), Marshall specimens for moisture stability testing were prepared with a diameter of 101.6 ± 0.25 mm and a height of 63.5 ± 1.3 mm.

**Figure 6:**
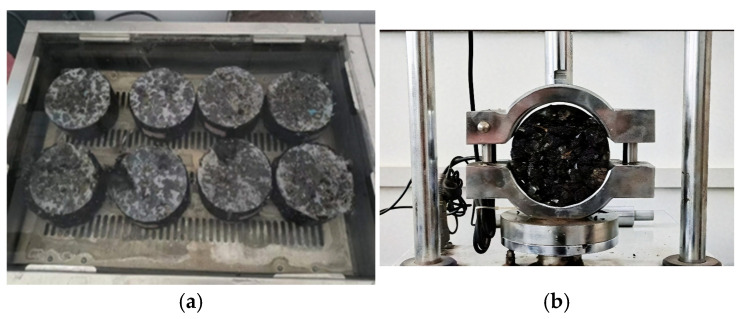
Water Bath Conditioning of Fiber-Reinforced Asphalt Mixtures and Marshall Stability Test: (**a**) Water Bath Conditioning of Asphalt Mixtures. (**b**) Marshall Stability Test.

(1) Immersed Marshall test: As shown in Figure 6a, the first set of Marshall specimens was conditioned in a thermostatic water bath (HH.w21.600 model, produced by Beijing Kewei Yongxing Instrument Co., Ltd., Beijing, China) at 60 °C for 40 min, whereas the second set was stored in the same bath at 60 °C for 48 h. As shown in Figure 6b, a Marshall stability tester (Model LX-AMS2, manufactured by Nanjing Leading Environmental Technology Co., Ltd., Nanjing, China) was then used to apply the load to both groups of fiber-reinforced SMA specimens. Marshall stability is measured for both specimen sets, and the corresponding stability ratio is derived to characterize the moisture damage resistance of the fiber-reinforced SMA mixture.

The formula used to calculate the retained stability is shown in Equation (6):

(6) $$RS=\frac{{MS}_{1}}{{MS}_{0}}$$

*RS*—Retained stability (%);

${MS}_{1}$—Stability after water immersion or freeze–thaw treatment (kN);

${MS}_{0}$—Stability under unconditioned (untreated) conditions (kN).

**Figure 7:**
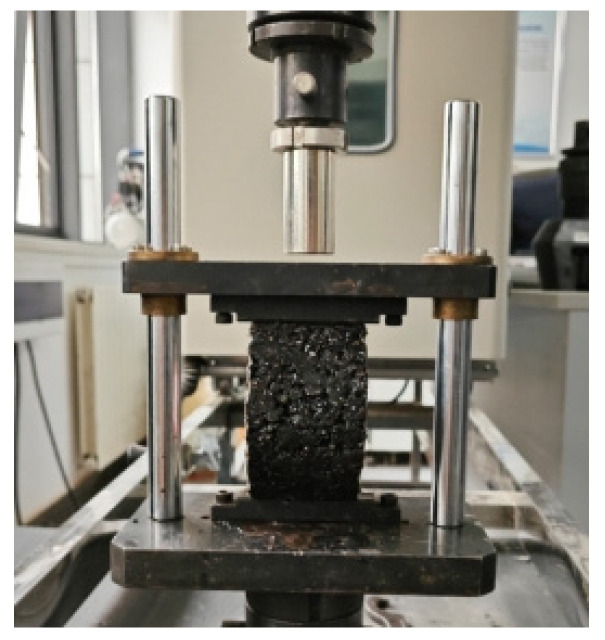
Splitting Test of Fiber-Reinforced Asphalt Mixtures.

(2) For the Freeze–Thaw Splitting test, the specimens are separated into two sets, each comprising at least four specimens. One group of Marshall specimens is subjected to vacuum saturation according to Standard Test Methods of Asphalt and Asphalt Mixture for Highway Engineering (JTG 3410-2025) T 0717-2025. Afterward, the specimens are tightly sealed in bags with approximately 10 mL of water and then stored in a freezer at −18 °C for 16 h. After freezing, both sets of specimens were immersed in a thermostatic water bath at 25 °C and conditioned for 24 h. As shown in Figure 7 and in compliance with specification T0716, a splitting tensile test was performed on the fiber-reinforced SMA mixture under a constant loading rate of 50 mm/min. The splitting tensile strengths of the two specimen sets were then calculated, and the corresponding freeze–thaw splitting strength ratio was determined.

For the Marshall freeze–thaw splitting test, the control specimens are employed to evaluate the splitting tensile strength $R_{T1}$; whereas the freeze–thaw-conditioned specimens are used to determine the splitting tensile strength $R_{T2}$. The calculation procedures for these splitting tensile strengths are given in Equations (7)–(9).

(7) $$R_{T1}=\frac{0.006287}{h1}{\times P}_{T1}$$

(8) $$R_{T2}=\frac{0.006287}{h2}\times P_{T2}$$

(9) $$TSR=\left(\frac{{\bar{R}}_{T2}}{{\bar{R}}_{T1}}\right)\times 100\%$$

*R*_*T*1_—splitting tensile strength (MPa) of an individual control specimen (without freeze–thaw cycles);

*R*_*T*2_—splitting tensile strength (MPa) of an individual freeze–thaw-conditioned specimen;

*P*_*T*1_—test load (N) of an individual control specimen;

*P*_*T*2_—test load (N) of an individual freeze–thaw-conditioned specimen;

*h*1—height (mm) of an individual control specimen;

*h*2—height (mm) of an individual freeze–thaw-conditioned specimen;

$\bar{R}$_*T*1_, $\bar{R}$_*T*2_—average splitting tensile strengths (MPa) of valid control and freeze–thaw-conditioned specimens, respectively;

*TSR*—the freeze–thaw splitting strength ratio.

#### 2.3.4. Viscoelastic Properties Test

**Figure 8:**
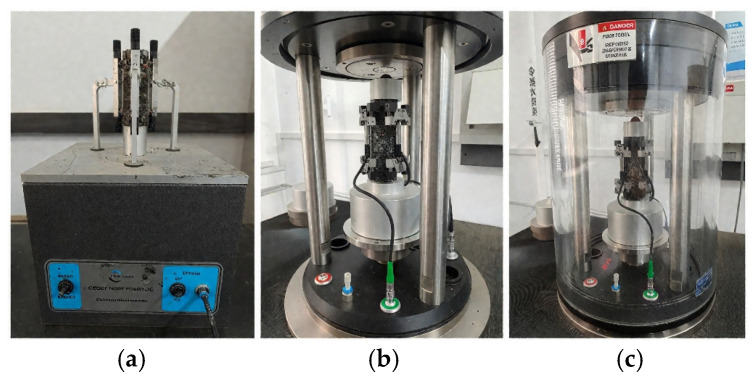
AMPT Testing Procedure: (**a**) Specimen Mounted with Positioning Pins. (**b**) Specimen Connected to Sensors. (**c**) Dynamic Modulus Test.

In this experiment, the AMPT Pro testing system (AMPT Pro Asphalt Mixture Performance Tester, produced by Guangzhou Oumei Dadi Instrument Equipment Co., Ltd., Guangzhou, China) was employed to determine the dynamic modulus and phase angle responses of SMA mixtures incorporating different types and contents of fibers, thereby investigating their viscoelastic mechanical behavior under dynamic loading conditions. The mixtures were first compacted using a gyratory compactor (AFG2C(S) model, Guangzhou Oumei Dadi Instrument Equipment Co., Ltd., Guangzhou, China) and then core-drilled and trimmed to obtain cylindrical specimens with a height of 110 mm and a diameter of 38 mm. The trimmed specimens were subsequently mounted in the dedicated clamping fixtures and positioned using the alignment pins supplied with the AMPT system, as illustrated in Figure 8. After mounting, the specimens were conditioned in a temperature-controlled chamber for 2 h. Dynamic modulus tests were conducted at three temperatures, namely 4 °C, 20 °C, and 40 °C. Following sensor installation, cyclic loading was applied in the triaxial chamber at four loading frequencies (0.1, 1, 10 and 25 Hz).

## 3. Results and Discussion

### 3.1. High-Temperature Stability

**Figure 9:**
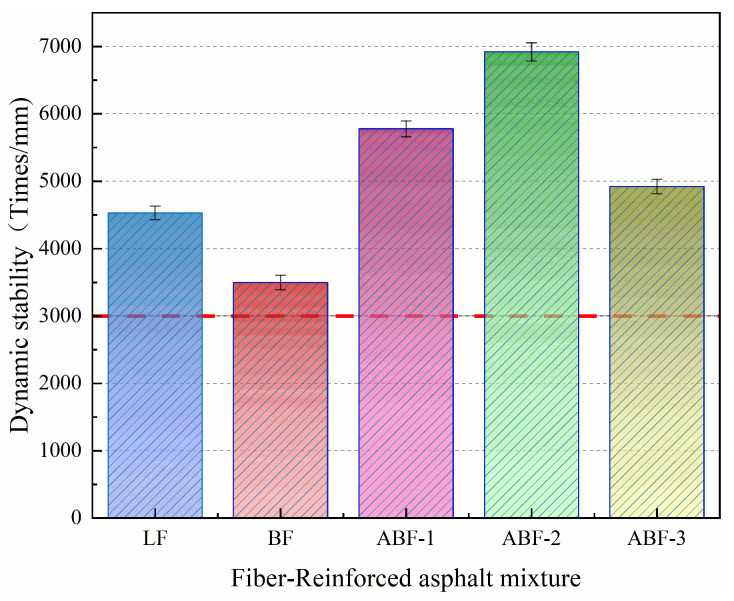
Dynamic Stability of Fiber-Reinforced SMA Mixtures.

As shown in Figure 9, the bamboo fiber-reinforced SMA mixture exhibits a dynamic stability that is 29.5% higher than that of the lignin fiber-reinforced SMA mixture. The combined use of bamboo and aramid fibers further improves the high-temperature stability, and Group ABF-2 achieved a dynamic stability of 6921 passes/mm, corresponding to increases of 97.7% relative to the lignin fiber mixture and 52.7% relative to the bamboo fiber mixture. Under a constant total fiber content, the dynamic stability of the asphalt mixture increased first and then decreased with an increasing aramid fiber proportion. This trend is mainly attributed to the synergistic effect between the high strength and modulus of aramid fiber and the oil-absorption and asphalt-stabilizing action of bamboo fiber, which enhances the structural stability and high-temperature stability of the mixture. However, when the aramid fiber proportion becomes excessively high (as in Group ABF-3), the reduced bamboo fiber content leads to more free asphalt, and aramid fiber agglomeration may occur, weakening the effective contribution of the fibers and resulting in a decrease in high-temperature stability.

The decline in high-temperature stability for ABF-3 may be attributed to aramid fiber agglomeration. As reported by He et al. [29], aramid fiber agglomeration is strongly affected by mixing time, temperature, and fiber content. Although direct microstructural evidence (e.g., X-ray CT or SEM image analysis) was not obtained in this study, the performance trend—dynamic stability increasing from ABF-1 to ABF-2 and then decreasing at ABF-3—is consistent with the hypothesis that excessive aramid fiber content exceeds the dispersion capacity of the dry-mixing method under the current mixing conditions (180 °C, 90 s dry mixing). Future work should employ quantitative dispersion analysis to directly validate this inference.

### 3.2. Low-Temperature Cracking Resistance

**Figure 10:**
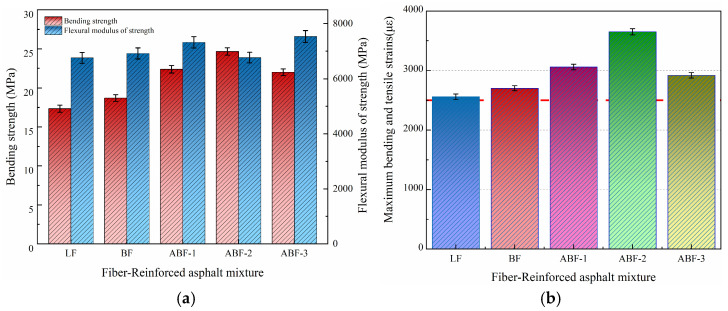
Results of low-temperature trabecular bending tests of asphalt mixtures: (**a**) asphalt mixture flexural strength and asphalt mixture flexural modulus results; (**b**) maximum bending tensile strain of the asphalt mixture.

As shown in Figure 10a, the low-temperature flexural beam test results indicate that the incorporation of hybrid fibers improves the low-temperature cracking performance of the SMA mixture. The flexural tensile strengths of the hybrid-fiber mixtures are generally higher than those of the single-fiber mixtures, and Group ABF-2 performed best, showing increases of 43.1% and 32.1% compared with the lignin–fiber and bamboo–fiber mixtures, respectively. Meanwhile, the hybrid-fiber mixtures exhibit overall lower flexural stiffness moduli than the single-fiber mixtures. In particular, ABF-2 maintains a high strength while presenting a lower stiffness modulus, indicating better flexibility and crack resistance, which helps mitigate brittle failure caused by low-temperature contraction. As shown in Figure 10b, the maximum flexural tensile strain of the hybrid-fiber groups is also higher, and ABF-2 increased by 42.6% relative to the lignin–fiber mixture and by 35.0% relative to the bamboo–fiber mixture. These improvements are mainly attributed to the high strength and modulus of aramid fibers, which redistribute stress at low temperatures, together with the adsorption and dispersion effects of bamboo fibers that promote the formation of a stable fiber–mastic structure and internal reinforcing network, thereby enhancing the strength, toughness, and deformation capacity of the mixture. Taken together, the lower flexural stiffness modulus and higher maximum flexural tensile strain of ABF-2 indicate that it has better deformation capacity before failure rather than merely increasing mixture stiffness, which contributes to its improved low-temperature cracking resistance.

Although direct fatigue crack propagation tests (e.g., four-point bending fatigue) were not performed in this study, previous research by Khan et al. [27] and Jia et al. [28] demonstrated that aramid fibers can significantly delay fatigue deterioration in asphalt mixtures. Given the high strength of aramid fibers and their effective stress transfer capability, it is plausible that the hybrid bamboo–aramid system could also exhibit enhanced fatigue resistance. However, direct confirmation through fatigue testing remains an important direction for future work.

### 3.3. Moisture Stability

**Figure 11:**
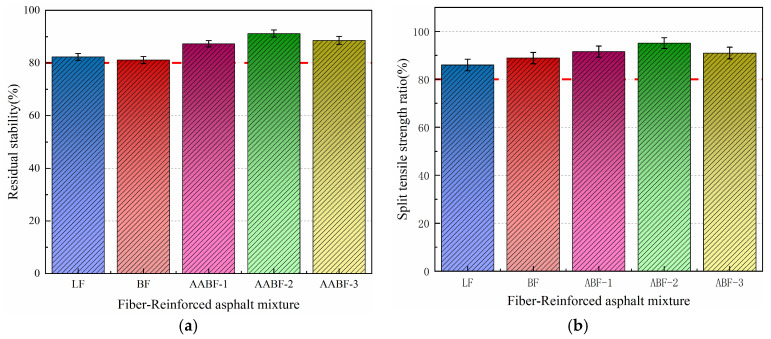
(**a**) Residual stability of asphalt mixtures. (**b**) Asphalt mixture split tensile strength ratio.

As illustrated in Figure 11a, the retained stability of the hybrid fiber-reinforced mixtures is noticeably higher than that of the single-fiber mixtures. In particular, Group ABF-2 increases by 10.8% and 12.5% compared with the lignin fiber and bamboo fiber mixtures, respectively. This improvement is mainly attributed to the synergistic effects of bamboo fibers in absorbing asphalt and aramid fibers in reinforcing the internal structure, which makes the mixture denser and strengthens asphalt–aggregate bonding, thereby reducing water intrusion and stripping.

As shown in Figure 11b, the freeze–thaw splitting strength ratios of all fiber-reinforced groups are higher than that of the control group, and ABF-2 exhibits the most pronounced improvement, increasing by 10.6% relative to the lignin fiber mixture and by 7.0% relative to the bamboo fiber mixture. These results indicate that hybrid-fiber reinforcement effectively enhances the moisture stability and freeze–thaw resistance of SMA mixtures, helping to extend pavement service life and reduce maintenance demand.

### 3.4. Viscoelastic Mechanical Properties

**Figure 12:**
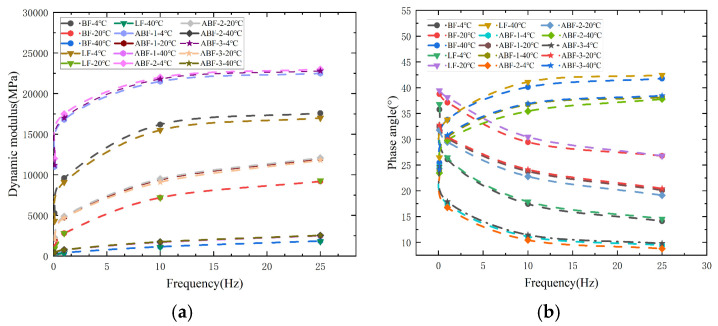
Dynamic Modulus and Phase Angle of Fiber-Reinforced Asphalt Mixtures: (**a**) dynamic modulus; (**b**) phase angle.

As illustrated in Figure 12a, the dynamic modulus is significantly affected by temperature and loading frequency. The dynamic modulus decreases markedly with increasing temperature, while it gradually increases as the loading frequency increases. Among all mixtures, Group ABF-2 exhibits a relatively high dynamic modulus, reaching approximately 23,000 MPa at 4 °C and 25 Hz, which is more than 30% higher than that of the single-fiber groups. This improvement is mainly attributed to the combined effects of bamboo and aramid fibers. Bamboo fiber can absorb and stabilize part of the asphalt, thereby improving the stability of the asphalt mastic, while aramid fiber provides additional reinforcement within the mixture and promotes stress transfer. These effects enable the hybrid fiber-reinforced mixture to maintain a more stable internal structure under repeated loading. Under high-temperature and low-frequency conditions, the differences among the groups become relatively small because the mixture response is more strongly affected by asphalt softening and the load-bearing role of the aggregate skeleton. However, under low-temperature conditions, ABF-2 still maintains a clear advantage, indicating its better adaptability to variations in temperature and frequency.

These dynamic modulus results can be further understood together with the low-temperature flexural beam results. Although ABF-2 shows a lower flexural stiffness modulus in the low-temperature bending test, this is not inconsistent with its higher dynamic modulus in the AMPT test. The AMPT dynamic modulus mainly reflects the stiffness response of the mixture under small-strain cyclic loading, whereas the low-temperature flexural beam test reflects the deformation and cracking behavior of the specimen under larger deformation until failure. Therefore, ABF-2 can maintain relatively high stiffness under cyclic loading while also showing improved deformation capacity and crack resistance under low-temperature bending. This indicates a better balance between stiffness and flexibility in the hybrid fiber system.

As illustrated in Figure 12b, the phase angle increases with temperature and decreases with frequency, reflecting the shift between viscous and elastic responses. The mixtures incorporating both aramid and bamboo fibers exhibit an overall improved viscoelastic response, suggesting a synergistic effect that enhances mechanical adaptability and durability under different service conditions.

It is worth noting that ABF-2 shows a relatively high dynamic modulus in the AMPT test but a lower flexural stiffness modulus in the low-temperature beam bending test. This apparent discrepancy is not contradictory but rather reflects the different testing conditions. The AMPT dynamic modulus measures the stiffness response under small-strain cyclic loading, where the material behaves within the linear viscoelastic range. In contrast, the low-temperature flexural beam test applies a monotonic loading at a high displacement rate until failure, inducing large deformation and crack propagation. The ability of ABF-2 to maintain relatively high stiffness under cyclic loading while exhibiting lower stiffness and higher strain capacity under monotonic failure loading indicates a favorable balance between stiffness and toughness, which is a hallmark of effective fiber reinforcement.

### 3.5. Mechanistic Discussion of the Synergistic Reinforcement

The observed performance improvements in the hybrid fiber-reinforced mixtures, particularly ABF-2, can be attributed to the complementary and synergistic actions of bamboo fiber and aramid fiber. On the one hand, bamboo fiber possesses a porous and irregular surface morphology, which provides abundant sites for asphalt absorption. This high oil absorption capacity allows bamboo fibers to adsorb and stabilize free asphalt within the mixture, reducing the amount of free asphalt that would otherwise contribute to plastic flow under high-temperature loading. Consequently, the asphalt mastic becomes more viscous and stable, enhancing the adhesion between the asphalt binder and the aggregate surface. This mechanism primarily contributes to improved high-temperature stability and moisture stability.

On the other hand, aramid fiber exhibits a straight, smooth, and high-strength morphology. With a tensile strength of 2900 MPa and a modulus substantially higher than that of bamboo fiber, aramid fibers act as a micro-reinforcement network within the mixture. They bridge across micro-cracks and micro-pores, transferring tensile stresses and preventing crack propagation. This bridging effect is particularly beneficial under low-temperature bending and cyclic loading conditions, where crack initiation and growth are the primary failure modes. In the hybrid system, these two mechanisms operate synergistically. The bamboo fibers create a stable, high-viscosity mastic that tightly binds the aggregate skeleton and reduces the mobility of aramid fibers, preventing their agglomeration.

In return, the aramid fibers provide the mechanical reinforcement that bamboo fibers alone cannot offer due to their lower intrinsic strength. This mutual enhancement explains why ABF-2 outperforms both single-fiber mixtures and the other hybrid ratios. However, when the aramid fiber content becomes excessively high, the reduced bamboo fiber content leads to insufficient asphalt adsorption, resulting in more free asphalt and a higher risk of aramid fiber agglomeration. This agglomeration creates stress concentration points and weakens the effective fiber network, explaining the decline in performance observed in ABF-3.

## 4. Conclusions

This study investigated the reinforcing effect of co-blended bamboo and aramid fibers on SMA-13 asphalt mixtures under a fixed total fiber content. Based on high-temperature stability, low-temperature cracking resistance, moisture stability, and viscoelastic characterization, the following conclusions can be drawn:

(1) The synergistic use of bamboo fiber and aramid fiber significantly enhances the comprehensive performance of SMA-13 mixtures. Bamboo fiber improves asphalt adsorption capacity and mastic stability, whereas aramid fiber provides high-strength reinforcement and facilitates stress transfer mechanisms. Their synergistic interaction jointly contributes to the structural stability and crack resistance of the mixture. Among the three tested proportions, ABF-2, containing 0.24% bamboo fiber and 0.06% aramid fiber (bamboo-to-aramid ratio of 4:1), exhibits the optimal performance, indicating that an appropriate mixing ratio plays a critical role in balancing mastic stability, fiber reinforcement, and deformation resistance.

(2) Compared with lignin fiber (LF) and pure bamboo fiber (BF) mixtures, ABF-2 demonstrates substantially improved pavement performance. Its dynamic stability reaches 6921 cycles/mm, representing increases of 97.7% and 52.7% over LF and BF, respectively. The flexural tensile strength increases by 43.1% and 32.1%, and the maximum flexural tensile strain increases by 42.6% and 35.0%, respectively. Furthermore, the retained stability increases by 10.8% and 12.5%, indicating enhanced resistance to moisture damage. Viscoelastic characterization further confirms that the bamboo–aramid hybrid fiber system increases the dynamic modulus and reduces the phase angle, endowing the mixture with a more pronounced elastic response and improved resistance to permanent deformation. These results demonstrate that the hybrid fiber system enhances the temperature–frequency adaptability and structural stability of SMA-13 mixtures under cyclic loading.

(3) In terms of engineering application, based on laboratory test results, ABF-2 is recommended for use in surface layers of high-grade highways, particularly in regions characterized by high temperatures, heavy traffic loads, and frequent rainfall. Although the incorporation of a low content (0.06%) of aramid fiber leads to a slight increase in material cost, the resulting improvement in performance over the pure bamboo fiber mixture is substantial, demonstrating a favorable cost-effectiveness balance.

(4) This study has several limitations. First, long-term aging tests have not been conducted, leaving the durability of the hybrid fiber system under oxidative aging and thermal cycling yet to be clarified. Second, a lack of field validation studies prevents the generalization of laboratory findings to real-world traffic and service environments. Third, fiber agglomeration is inferred solely from macroscopic performance degradation, without direct observation using microstructural characterization techniques. Finally, due to equipment constraints, fatigue crack propagation tests were not performed. These limitations will be addressed in future research.

Overall, the co-blending of bamboo and aramid fibers offers an effective approach for improving the comprehensive performance of SMA-13 mixtures. Future research should further evaluate the long-term durability, field performance, and microstructural characteristics of bamboo–aramid hybrid fiber-reinforced mixtures and explore their potential application in sustainable and low-carbon pavement construction.

## Figures and Tables

**Table 1:** Summary of key parameters and reported effects of bamboo and aramid fibers in asphalt mixtures from the literature.

Fiber Type	Recommended Length (mm)	Recommended Content (%)	Key Reported Effects	Representative References
Bamboo fiber	3–9	0.2–0.4	Asphalt adsorption, high-temperature stability, low-temperature crack resistance, anti-aging	Sheng et al. [18], Jia et al. [19,21], Cao et al. [20]
Aramid fiber	3–12	0.03–0.1	Fatigue resistance, dynamic modulus enhancement, moisture damage resistance, aging delay	Saliani et al. [25], Badeli et al. [26], Khan et al. [27], Jia et al. [28]

**Table 2:** Physical Parameters of Bamboo and Aramid Fibers.

Parameter	Unit	Value/Bamboo Fibers	Value/Aramid Fibers
Density	g/cm^3^	0.779	1.440
Diameter	µm	35	12
Length	mm	6	6
Oil Absorption Rate	g/g	4.95	1.69
Moisture Content	%	9.5	7.2
Mass Loss	%	8.1	1.09
Tensile Strength	MPa	600	2900

**Table 3:** Technical Properties of Coarse Aggregate.

Test Item		Specification Requirement	Test Result
Aggregate Crushing Value (%)		≤20	12.6
Los Angeles Abrasion Loss (%)		≤24	13.3
Apparent Relative Density (g/cm^3^)	10~15 mm	≥2.6	2.873
	5~10 mm	≥2.6	2.779
	3~5 mm	≥2.6	2.788
Water Absorption (%)	10~15 mm	≤2.0	0.63
	5~10 mm	≤2.0	0.69
	3~5 mm	≤2.0	0.71
Flaky and Elongated Particle Content (%)		≤15	6.2
Clay Content (%)	10~15 mm	≤1	0.31
	5~10 mm	≤1	0.42
	3~5 mm	≤1	0.36

**Table 4:** Technical Properties of Fine Aggregate.

Test Item	Technical Requirement	Test Result
Apparent Relative Density (g/cm^3^)	≥2.5	2.878
Water Absorption (%)	≤3.0	1.31
<0.075 mm Particles Passing (%)	≤1.0	0.17

**Table 5:** Technical Properties of Mineral Powder.

Index		Specification Requirement	Test Result
Apparent Density (g/cm^3^)		≥2.5	2.799
Hydrophilic Coefficient		≤1	0.84
Particle Size Distribution (%)	<0.6 mm	100	100
	<0.15 mm	90~100	95.2
	<0.075 mm	75~100	83.1
Appearance		No Agglomeration or Lumps	Qualified

**Table 6:** Technical Properties of SBS Modified Asphalt.

Test Item	Unit	Specification Value	Test Result
Penetration (25 °C, 5 s, 100 g)	0.1 mm	80–100	88.0
Softening Point	°C	≮50	61
Ductility (5 °C)	cm	≮40	46
Flash Point	°C	≮230	253
Dynamic Viscosity (60 °C)	Pa·s	≮160	207
Density (15 °C)	g/cm^3^	-	1.013
Elastic Recovery Rate	%	≮60	93

**Table 7:** Fiber Content in Asphalt Mixtures.

Group Name	Bamboo Fiber Content (%)	Aramid Fiber Content (%)	Proportion
LF (Lignin Fiber 0.3%)	0	0	-
BF	0.3	0	-
ABF-1	0.27	0.03	9:1
ABF-2	0.24	0.06	4:1
ABF-3	0.21	0.09	7:3

Note: The following abbreviations are used throughout this paper: LF = lignin fiber mixture (0.3% lignin fiber); BF = bamboo fiber mixture (0.3% bamboo fiber); ABF-1 = hybrid mixture with 0.27% bamboo + 0.03% aramid (9:1 ratio); ABF-2 = hybrid mixture with 0.24% bamboo + 0.06% aramid (4:1 ratio); ABF-3 = hybrid mixture with 0.21% bamboo + 0.09% aramid (7:3 ratio). The numbers 1, 2, and 3 indicate increasing aramid fiber proportion.

**Table 8:** Marshall Performance Indicators of ABF-3 composite fiber SMA-13 Asphalt Mixtures.

Asphalt–Aggregate Ratio (%)	Theoretical Maximum Relative Density (g/cm^3^)	Bulk Density (g/cm^3^)	Air Voids (%)	Voids in Mineral Aggregate (VMA, %)	Asphalt Saturation (%)	Stability (kN)	Flow Value (mm)
5.5	2.689	2.464 ± 0.012	6.32 ± 0.21	16.71 ± 0.18	70.45 ± 0.9	12.32 ± 0.31	2.46 ± 0.08
6.0	2.622	2.532 ± 0.010	5.11 ± 0.18	16.13 ± 0.15	73.63 ± 1.2	13.41 ± 0.28	2.58 ± 0.07
6.5	2.560	2.621 ± 0.011	4.27 ± 0.15	15.92 ± 0.16	75.18 ± 1.0	14.44 ± 0.35	2.63 ± 0.10
7.0	2.526	2.549 ± 0.012	3.44 ± 0.14	15.31 ± 0.17	78.61 ± 0.9	12.21 ± 0.33	2.78 ± 0.07

**Table 9:** Marshall Performance Indicators of Fiber-Modified SMA-13 Asphalt Mixtures.

Group	Asphalt–Aggregate Ratio (%)	Bulk Density (g/cm^3^)	Air Voids (%)	Voids in Mineral Aggregate (VMA, %)	Asphalt Saturation (%)	Stability (kN)	Flow Value (mm)
LF	6.6	2.486 ± 0.011	6.15 ± 0.14	16.50 ± 0.18	71.20 ± 1.1	12.83 ± 0.28	2.50 ± 0.07
BF	6.4	2.519 ± 0.009	5.25 ± 0.18	16.35 ± 0.13	72.16 ± 0.9	13.16 ± 0.29	2.55 ± 0.09
ABF-1	6.6	2.451 ± 0.013	6.10 ± 0.19	16.67 ± 0.19	70.62 ± 0.9	12.53 ± 0.31	2.47 ± 0.05
ABF-2	6.5	2.529 ± 0.012	5.30 ± 0.21	16.27 ± 0.18	73.65 ± 1.2	13.26 ± 0.29	2.63 ± 0.10
ABF-3	6.3	2.471 ± 0.011	6.21 ± 0.19	16.44 ± 0.17	71.53 ± 0.8	12.63 ± 0.30	2.49 ± 0.09

**Table 10:** Results of Cantabro Raveling Test.

Mixture Type	$m_{0}$ (g)	$m_{1}$ (g)	∆S (%)	Specification Requirement
LF	1257.7	1191.7	5.25	ΔS ≤ 15%
BF	1250.5	1194.2	4.55	
ABF-1	1244.2	1185.1	4.75	
ABF-2	1250.3	1184.9	4.77	
ABF-3	1243.6	1183.1	4.91	

**Table 11:** Results of Schellenberg Draindown Test.

Test Temperature (°C)	Mixture Type	Asphalt–Aggregate Ratio (%)	$m_{0}$ (g)	$m_{1}$ (g)	$m_{2}$ (g)	∆m (%)	Specification Requirement
185 °C	LF	6.6	210	1449.4	210.9	0.07	∆m ≤ 0.1%
	BF	6.4	210	1447.5	210.6	0.05	
	ABF-1	6.6	210	1453.2	210.6	0.05	
	ABF-2	6.5	210	1448.6	210.6	0.06	
	ABF-3	6.3	210	1462.5	210.7	0.06	

## Data Availability

The original contributions presented in this study are included in the article. Further inquiries can be directed to the corresponding author.
